# The enhanced beam sweeping algorithm for DOA estimation in the hybrid analog-digital structure with nested array

**DOI:** 10.1038/s41598-022-04792-0

**Published:** 2022-02-22

**Authors:** Shujun Ye, Guanhao Liu, Yan Zhou, Bo Dang

**Affiliations:** 1 School of Information Science and Technology, Northwest University, Xian, 710127 China; 2 School of Electronic Engineering, Xian Shiyou University, Xian, 710065 China

**Keywords:** Electrical and electronic engineering, Information technology

## Abstract

As an emerging technology, the hybrid analog-digital structure has been considered for use in future millimeter-wave communications. Although this structure can reduce the hardware cost and power consumption considerably, the spatial covariance matrix (SCM), as the core of subspace-based direction of arrival (DOA) estimation, cannot be obtained directly. Previously, the beam sweeping algorithm (BSA) has been found effective for reconstructing the spatial covariance matrix and realizing DOA estimation by forming the beams to difference directions. However, it is computationally intractable owing to the high-dimensional matrix operation. To address this problem and improve the DOA estimation performance, this paper applies the nested array to the hybrid analog-digital structure and proposes the enhanced BSA (EBSA) for DOA estimation. By deleting a large number of redundant elements exist in the SCM to be reconstructed, the computational cost can be considerably reduced. Also, the nested array can offer high degrees of freedom. Finally, simulation experiments are conducted to verify the performance of EBSA. The results indicate that the proposed EBSA is better than the state-of-the-art method in terms of estimation accuracy and computational cost.

## Introduction

Direction of arrival (DOA) estimation has been widely applied in wireless communication, radar and so on^1–3^. By subspace DOA estimation methods like multiple signal classification (MUSIC) algorithms, the number of signals that can be estimated with an *N* elements uniform linear array is $N-1$^4^. To effectively save the cost of antennas and achieve improved DOA estimation performance, sparse array structures have received much attention. Compared with uniform linear array, the sparse array can provide the same degrees of freedom (DOFs) with reduced number of antennas. Among the well-known sparse arrays, the nested array is of considerable interest to researchers. Nested array is obtained by systematically nesting several uniform linear arrays to achieve $O\left( {N^{{2}} } \right)$ the degrees of freedoms with only $O\left( {N } \right)$ antennas. Considering superior characteristics of the nested array such as explicit expression for the array element position, it is considered as the research objective of the present study^5^ added sensors to the original nested array and improved the performance of the original scheme. In this regard^6^ established a super nested array to reduce the mutual coupling. Moreover,^7^ splited the dense uniform linear array in nested array into two or four parts and proposed the augmented nested array. Further investigations showed that the idea of one-dimensional sparse array design can be extended to the two-dimensional (2D) sparse array for 2D DOA estimation. In this regard,^8^ designed the 2D nested array. Shu et al.^9^ extended the nested array to the near-field source localization and the full co-array aperture of parallel nested array in 2D direction finding was illustrated. The extensions of nested array to DOA estimation with high-order cumulants are considered in^10–12^.

To reduce the hardware cost, the hybrid analog-digital structure has been designed for the millimeter-wave communications^13–15^. In this structure, the signal received by multiple antennas is combined and then fed to the digital receiver. Therefore, the spatial covariance matrix (SCM) is unavailable and the subspace-based DOA estimation algorithm cannot be adopted. Chuang et al.^16^ proposed an augmented beam-space approach (H-MUSIC) to solve the problem. Ulteriorly, the preprocessing scheme is extended to the fully-connected hybrid architecture in^17^. To obtain the DOA information, an approach (HDAPA) based on the maximum received power was explored in^18^. By finding the consistent sign of the cross-correlation of subarray gains, a robust and unambiguous DOA estimation algorithm was proposed in^19^. To eliminate the restriction by the Rayleigh limitation in HDAPA, a novel and simple beam sweeping algorithm (BSA) was proposed for SCM reconstruction in^20,21^. By forming different beams, the SCM can be reconstructed based on the solutions to linear equations. Then, super-resolution DOA estimation can be realized by the MUSIC algorithm.

Though the BSA proposed in^20^ is effective, it requires $N^2$-dimensional (*N* is the number of antennas connected to one radio frequency chain) matrix inversion for reconstructing the SCM, which is computationally intractable. To address this problem and improve the DOA estimation performance for the original BSA, this paper applies nested array to the hybrid analog-digital structure and proposes the enhanced BSA (EBSA) for DOA estimation. By deleting a large number of redundant elements exist in the SCM to be reconstructed, the computational overhead in solving the linear equations can be reduced. In addition, the degrees of freedoms for DOA estimation can also be improved by deploying the antenna array as nested array, thus achieving more resolved sources and better DOA estimation accuracy. Simulation experiments are conducted to verify the performance of EBSA.

## Signal model

**Figure 1:**
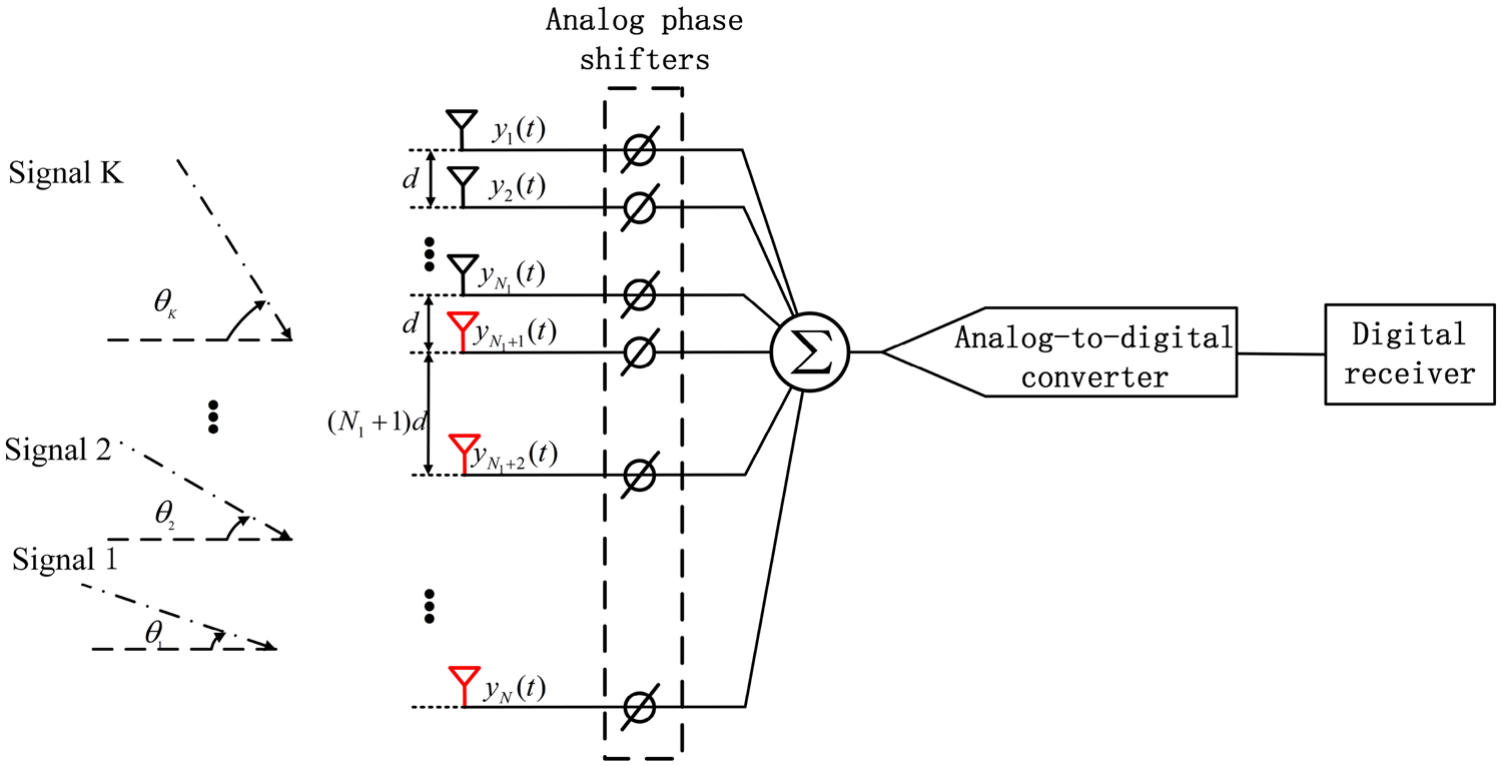
The hybrid analog-digital structure with nested array.

The signal model of the hybrid analog-digital structure with nested array is shown in Fig. 1, where one radio frequency chain is connected to multiple antennas. The nested array consists of two subarrays, of which subarray 1 contains $N_1$ antennas, while subarray 2 has $N_2$, hence a total of $N = N_1 + N_2$ antennas is considered. Antenna positions can be expressed as

1 $$\begin{aligned} P&= \left\{ 0:(N_1 - 1)\right\} d \cup \left\{ (1:N_2 )(N_1 + 1) - 1\right\} d = \left\{ {p_1 d,\;p_2 d,\ldots ,\;p_N d} \right\} , \end{aligned}$$

where $d = \lambda /2$ represents the half-wavelength spacing. The received signal at each antenna is represented as

2 $$\begin{aligned} {\mathbf{y}}(t)&= [y_1 (t),y_2 (t), \ldots ,y_N (t)]^T = \sum \limits _{k = 1}^K {\mathbf{a}} (\theta _k )s_k (t) + {\varvec{\varepsilon }}(t), \end{aligned}$$

where ${\mathbf{a}}(\theta _k ) = [{\mathop {\mathrm{e}}\nolimits } ^{ - j2\pi p_1 d\sin (\theta _k )/\lambda )} , \ldots ,{\mathop {\mathrm{e}}\nolimits } ^{ - j2\pi p_N d\sin (\theta _k )/\lambda } ]^T$ represents the steering vector for the incident angle of $\theta _k$, $s_{k}(t)$ denotes K narrow-band signals impinging from far field onto the array, ${\varvec{\varepsilon }}(t)$ denotes the Gaussian white noise and $[ \bullet ]^T$ represents the transpose. After **y**(t) is obtained, the spatial covariance matrix can be calculated as

3 $$\begin{aligned} {\mathbf{R}} = E[{\mathbf{y}}(t){\mathbf{y}}^H (t)] = \sum \limits _{k = 1}^K {\sigma _k^2 {\mathbf{a}}(\theta _k ){\mathbf{a}}^H (\theta _k )} + \sigma _n^2 {\mathbf{I}}_N, \end{aligned}$$

where ${\mathbf{I}}_N$ is an $N \times N$ identity matrix, $\sigma _n^2$ is the noise power, $\sigma _k^2$ is the power of the *k*th source and $[ \bullet ]^H$ represent the transpose conjugate. However, since one radio frequency chain is connected to multiple antennas in the hybrid analog-digital structure, ${\mathbf{y}}(t)$ is unknown to the digital receiver. Denoting the temporal domain sampling period as $T_s$, the signal summation obtained at the digital receiver is expressed as

4 $$\begin{aligned} c_q [l] = {\mathbf{a}}^H (\varphi _q ){\mathbf{y}}[l], l = 1,2, \ldots ,L, q = 1,2, \ldots ,Q, \end{aligned}$$

where ${\mathbf{a}}(\varphi _q ) = [{\mathop {\mathrm{e}}\nolimits } ^{ - j2\pi p_1 d\sin (\varphi _q )/\lambda )} , \ldots ,{\mathop {\mathrm{e}}\nolimits } ^{ - j2\pi p_N d\sin (\varphi _q )/\lambda } ]^T$ is the steering vector for the *q*th predetermined DOA angle, and *L* is the number of samples, ${\mathbf{y}}[l] = {\mathbf{y}}(lT_s )$ and $c[l] = c(lT_s )$. When *L* is large enough, the average power of $c_q[l]$ can be represented as

5 $$\begin{aligned} P_q&= \frac{1}{L}\sum \limits _{l = 1}^L {c_q [l]} c_q^H [l] = {\mathbf{a}}^H (\varphi _q )\frac{1}{L}\sum \limits _{l = 1}^L {{\mathbf{y}}[l]} {\mathbf{y}}^H [l]{\mathbf{a}}(\varphi _q ) = {\mathbf{a}}^H (\varphi _q ){\mathbf{Ra}}(\varphi _q ). \end{aligned}$$

To reconstruct ${\mathbf{R}}$ from $P_q$ by BSA, (5) is firstly vectorized as

6 $$\begin{aligned} P_q &= \mathrm{vec}[{\mathbf{a}}^H (\varphi _q ){\mathbf{Ra}}(\varphi _q )] = {\mathbf{a}}^T (\varphi _q ) \otimes {\mathbf{a}}^H (\varphi _q ) \mathrm{vec}({\mathbf{R}})= [{\mathbf{a}}(\varphi _q ) \otimes {\mathbf{a}}^ * (\varphi _q )]^T {\mathbf{r}}, \end{aligned}$$

where ${\mathbf{r}} = \mathrm{vec}({\mathbf{R}})$, $\mathrm{vec}\{ {\bullet }\}$ denotes the straightening of a matrix into a vector and $\otimes$ denotes the Kronecker product operation. Denoting ${\mathbf{a}}_q = {\mathbf{a}}(\varphi _q ) \otimes {\mathbf{a}}^ * (\varphi _q )$, (6) can be further extended as

7 $$\begin{aligned} {\mathbf{Ar }} = [P_1,\;P_2,\ldots ,\;P_Q]^T, \end{aligned}$$

where ${\varvec{A}}=\left( {\varvec{a}}_{1}, {\varvec{a}}_{2}, \ldots , {\varvec{a}}_{Q}\right) ^{\mathrm {T}}$. The vector ${\mathbf{r}}$ can be easily solved through the linear equations, and ${\mathbf{R}}$ can also be reconstructed based on the solution of ${\mathbf{r}}$. After ${\mathbf{R}}$ is obtained, the MUSIC algorithm can be applied to the hybrid analog-digital structure to achieve the DOA of each signal. Though the original BSA is straightforward and effective, it is computationally intractable due to the full-dimension matrix inversion in solving (7). In fact, by examining the elements in ${\mathbf{R}}$, it can be found that there are many redundant elements in ${\mathbf{R}}$, which can be eliminated to save the computational cost. Providing low computational cost and high degrees of freedoms for DOA estimation, the proposed EBSA based on the hybrid analog-digital structure with nested array will be described in detail as follows.

## Algorithm implementation

Based on (3), ${\mathbf{R}}$ can be expressed as

8 $$\begin{aligned} {\mathbf{R}}{{ = }}\left[ {\begin{array}{*{20}c} {r_{1 ,1 } } & {r_{1 ,2 } } & \cdots & {r_{1 ,N } } \\ {r_{2 ,1 } } & {r_{2 ,2 } } & \cdots & {r_{2 ,N } } \\ \vdots & \vdots & \ddots & \vdots \\ {r_{N ,1 } } & {r_{N ,2 } } & \cdots & {r_{N ,N } } \\ \end{array}} \right] \end{aligned}$$

where ${r_{n_1 ,n_2 }}$ is computed as

9 $$\begin{aligned} r_{{n_1} ,{n_2} }&= {\gamma }_{{p_{n_1} , p_{n_2} }} = \frac{1}{L}\sum \limits _{l = 1}^L {y_{n_1} [l]y_{n_2}^ * [l]}\\&=\sum \limits _{k = 1}^K {\sigma _k^2 e^{ - j2\pi \frac{{(p_{n_1} - p_{n_2} )d\sin (\theta _k )}}{\lambda }} } + \sigma _n^2 \delta (n_1 - n_2), 1 \le n_1,n_2 \le N, \end{aligned}$$

where $$\delta (n_1 - n_2) = \left\{ {\begin{array}{*{20}c} {1,\quad n_1 = n_2} \\ {0,\quad n_1 \ne n_2} \\ \end{array}} \right.$$. By carefully analyzing the expression of ${\mathbf{R}}$ in (8) and (9), it can be found that although there are $N^2$ elements in ${\mathbf{R}}$, the number of distinct elements is only $2N_2 (N_1 + 1) - 1$, which is definitely smaller than $N^2$. By extracting all the distinct elements and arranging them into a vector, a column vector that consists of these elements can be represented as

10 $$\begin{aligned} {\bar{\mathbf{r}}} =&\left[ {{\gamma }_{{0 , M - 1 }} ,{\gamma }_{{1 , M - 1 }} ,\ldots , {\gamma }_{{M - 1 , M - 1 }} ,} \right. \left. {{\gamma }_{{M - 1 , M - 2 }} , \ldots ,{\gamma }_{M - 1 , 0} } \right] ^T, \\ \end{aligned}$$

where $M = N_2 (N_1 + 1)$. It is noted that $\mathbf{r}$ can be deduced from ${\bar{\mathbf{r}}}$ by the following relation

11 $$\begin{aligned} {\mathbf{r}} = {\mathbf{J}{\bar{\mathbf{r}}}}, \end{aligned}$$

where ${\mathbf{J}}$ is a selecting matrix with dimension of $N^2 \times [2N_2 (N_1 + 1) - 1]$. It can be expressed as

12 $$\begin{aligned} \mathbf{J} = \left[ {\begin{array}{*{20}c} {{\mathbf{O}}_{p_N - p_1 } } & {\mathbf{V}} & {{\mathbf{O}}_{p_1 } } \\ {{\mathbf{O}}_{p_N - p_2 } } & {\mathbf{V}} & {{\mathbf{O}}_{p_2 } } \\ \vdots & {\mathbf{V}} & \vdots \\ {{\mathbf{O}}_{p_N - p_N } } & {\mathbf{V}} & {{\mathbf{O}}_{p_N } } \\ \end{array}} \right] , \end{aligned}$$

where ${{\mathbf{O}}_m }$ is the all-zero matrix with dimension of $N \times m$, ${\mathbf{V}} = [{\mathbf{l}}_1 ,{\mathbf{l}}_2 ,\ldots ,{\mathbf{l}}_N ]^T$, and ${\mathbf{l}}_n$ is an $N_2 (N_1 + 1) \times 1$ vector of all zeros except a 1 at the $(p_n + 1)$th position. Now (7) can be rewritten as

13 $$\begin{aligned} {{{\bar{\mathbf{A}}}{\bar{\mathbf{r}}} }} = {\mathbf{p}}, \end{aligned}$$

where ${{{\bar{\mathbf{A}}}}}={\mathbf{AJ}}$ and ${\mathbf{p}} = [P_1 ,P_2 ,\ldots ,P_Q ]^T$. Since there are $2N_2 (N_1 + 1) - 1$ unknowns in ${\bar{\mathbf{r}}}$, at least $2N_2 (N_1 + 1) - 1$ independent linear equations are required to solve (13). Therefore, the number of predetermined DOA angles should be no smaller than $2N_2 (N_1 + 1) - 1$, namely $Q \ge 2N_2 (N_1 + 1) - 1$. In addition, the solution of (13) is likely to be ill-conditioned because ${{{\bar{\mathbf{A}}}}}$ may be rank-deficient. According to^20^, the ill-conditioned solution can be avoided by diagonal loading. Defining $\sigma ^2$ to be the diagonal loading coefficient, ${\bar{\mathbf{r}}}$ can be calculated as

14 $$\begin{aligned} {\bar{\mathbf{r}}} = (\bar{\mathbf{A}}^H \bar{\mathbf{A}}{{ + }}\sigma ^{{2}} \bar{\mathbf{I}})^{ - 1} \bar{\mathbf{A}}^H {\mathbf{p}}, \end{aligned}$$

where $\bar{\mathbf{I}}$ indicates the $[2N_2 (N_1 + 1) - 1] \times [2N_2 (N_1 + 1) - 1]$ identity matrix and $[ \bullet ]^{-1}$ denotes the inverse operation of a matrix. In the original BSA, the dimension for the matrix inversion is $N^2 \times N^2$. By contrast, the dimension for the matrix inversion in EBSA is $[2N_2 (N_1 + 1) - 1] \times [2N_2 (N_1 + 1) - 1]$, which indeed reduces the computational cost for solving the linear equations. Finally, the equivalent SCM can be reconstructed as

15 $$\begin{aligned} {{{\bar{\mathbf{R}}}}}(n_1 ,\;n_2 ) = {\gamma }_{{p_{n_1} , p_{n_2} }},\quad 1 \le n_1 ,\;n_2 \le N. \end{aligned}$$

As for the proposed EBSA, the maximum number of signals that can be realized on DOA estimation is $N_2 (N_1 + 1) - 1$, while it is $N-1$ for the original BSA based on the uniform linear array with *N* elements. After ${{{\bar{\mathbf{R}}}}}$ is obtained, the MUSIC algorithm can be applied, and the DOA estimation in the hybrid analog-digital structure with nested array is realized.

Now, the computational cost of each algorithm for reconstructing the SCM is analyzed. The multiplication and division number (MDN) is used to measure the computational cost. Let ${\bar{N}} = 2N_2 (N_1 + 1) - 1$, thus the total MDN in solving (13) for reconstructing ${{{\bar{\mathbf{R}}}}}$ can be expressed as

16 $$\begin{aligned}{\bar{N}} \times Q \times {\bar{N}} + \frac{2}{3}({\bar{N}} )^3 + {\bar{N}} \times {\bar{N}} \times Q \ + {\bar{N}} \times Q . \end{aligned}$$

As described in^5^, by considering the optimal allocation of nested array, (16) can be further simplified as

17 $$\begin{aligned} \left\{ {\begin{array}{l} \begin{array}{l} \frac{{N^6 }}{{12}} + \frac{{N^5 }}{2} + \frac{{(1 + Q)N^4 }}{2} + \frac{{(6Q - 4)N^3 }}{3} + \frac{{(Q - 2)N^2 }}{2} + (2 - 3Q)N + Q - \frac{2}{3},\quad N\; {\mathrm{is}} \;{\mathrm{even}} \\ \end{array} \\ \begin{array}{l} \frac{{N^6 }}{{12}} + \frac{{N^5 }}{2} + \frac{{(3 + 2Q)N^4 }}{4} + \frac{{(6Q - 1)N^3 }}{3} + \frac{{(4Q - 3)N^2 }}{4} + \frac{{(1 - 4Q)N}}{2} + \frac{Q}{2} - \frac{1}{{12}},\quad N\; {\mathrm{is}} \;{\mathrm{odd}} \\ \end{array} \\ \end{array}} \right. . \end{aligned}$$

In BSA, the total MDN for reconstructing the SCM is

18 $$\begin{aligned}N^2 \times Q \times N^2 + \frac{2}{3}N^6 + N^2 \times N^2 \times Q + N^2 \times Q \times 1 = \frac{2}{3}N^6 + 2QN^4 + QN^2 \end{aligned}$$

As the compassion of (18) and (17), it is obvious that when the antenna number is large, the computational cost of EBSA for spatial covariance matrix reconstruction is greatly reduced.

## Simulation results

**Figure 2:**
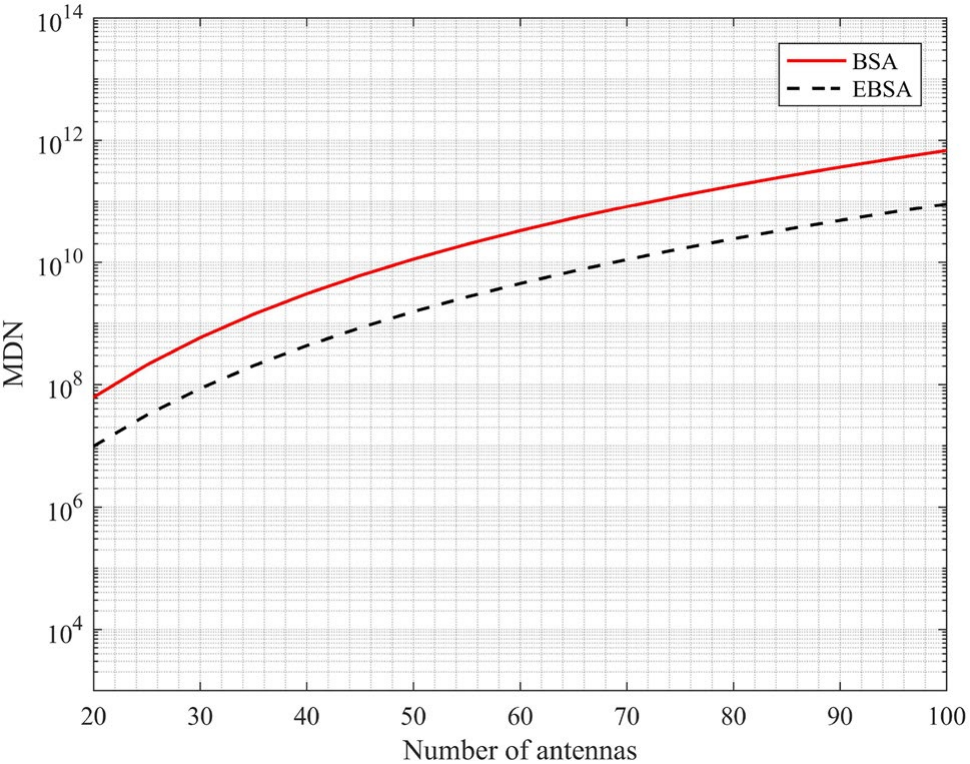
Computational cost comparison.

The advantage of the proposed algorithm in computational cost for reconstructing the SCM is first considered particularly. Without special declaration, EBSA refers to the EBSA based on the hybrid analog-digital structure for nested array with the optimal allocation as^5^, while BSA refers to the BSA based on the hybrid analog-digital structure for uniform linear array as in^20^. To ensure the accuracy of the estimation, the number of predetermined DOA angles is set to $N(N-1)$ when the number of antennas is set to *N* for both EBSA and BSA. It can be seen from Fig. 2 that the computational cost of EBSA is always less than that of BSA. Especially, for a large number of antennas, the computational cost of EBSA can be greatly reduced. Ulteriorly, the reconstruction time for EBSA and BSA is compared in Table 1. In this comparison experiment, 5 signals with SNR (signal to noise ratio) of 0 dB uniformly distributed from $- 50^ \circ$ to $50^ \circ$ are considered, while the number of samples is 1500, the diagonal coefficient $\sigma ^2 = 1$. The simulation experiment is implemented with Intel(R) Core(TM) i7-4790 3.6GHz CPU and 12GB RAM by running the MATLAB codes in the same environment. Results show the time for covariance matrix reconstruction is effectively saved by the proposed EBSA.

**Table 1:** Simulation time of covariance matrix reconstruction.

Number of antennas	BSA	EBSA
10	0.0010s	0.0004s
20	0.0021s	0.0008s
30	0.1646s	0.0536s
40	1.0548s	0.2370s
50	4.3388s	0.9090s
60	13.2742s	2.8061s
70	31.6204s	8.6792s
80	75.3630s	15.2489s
90	136.6410s	29.7198s
100	261.1464s	54.9779s

In the following simulations, 6 antennas are deployed in both nested array and uniform linear array. $N_1=N_2=3$ is set to ensure the optimal allocation of nested array. For both EBSA and BSA, $Q =5N_2 (N_1 + 1) - 1 = 59$ predetermined DOA angles are evenly selected from $- 90^ \circ$ to $90^ \circ$. To avoid the generation of ill-conditioned solution, the diagonal coefficient $\sigma ^2 = 1$ is selected. All the signals to be estimated are uniformly distributed from $- 50^ \circ$ to $50^ \circ$.

**Figure 3:**
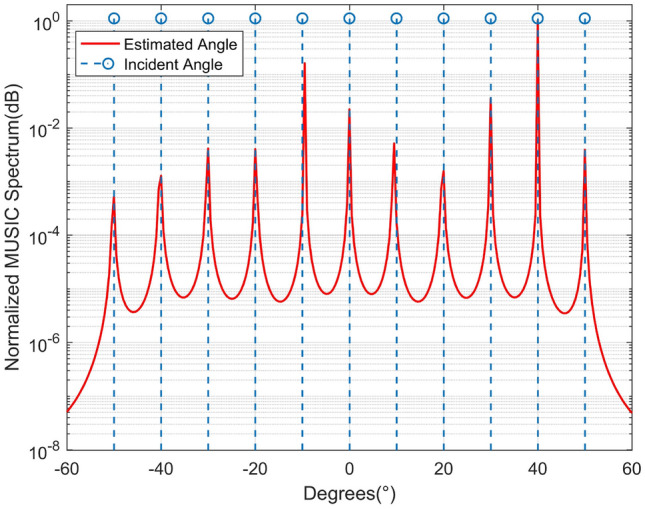
DOA estimation spectrum for EBSA.

The second simulation illustrates the MUSIC spectrum of EBSA, and the result is shown in Fig. 3. There are 11 signals with SNR of 0 dB, and the number of samples is 1500. The blue dot lines in Fig. 3 exhibit the true incident angle of each signal. Given 6 antennas, it can be seen clearly that EBSA accurately resolves all the 11 signals. It is worth noting that the dimension of the reconstructed SCM in BSA is smaller than 11 and the MUSIC spectrum of BSA is not exist.

**Figure 4:**
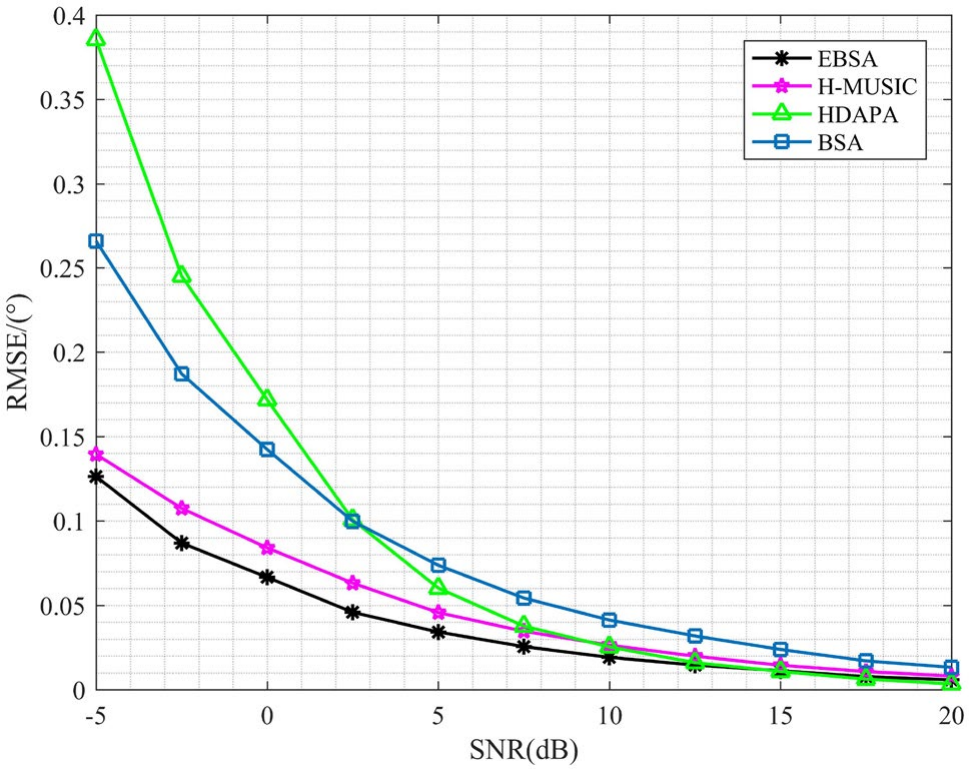
RMSE curves with different SNR .

**Figure 5:**
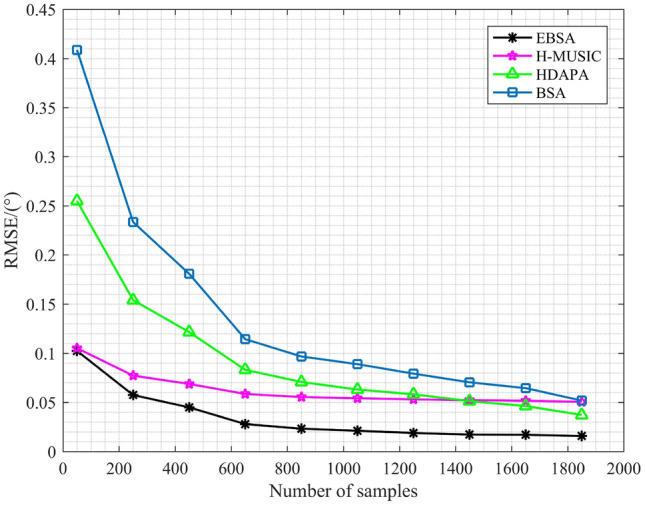
RMSE curves with different number of samples.

Now a set of simulations are conducted to compare the estimation accuracy of EBSA, H-MUSIC^16^, HDAPA ^18^and BSA. The estimation root mean-square error (RMSE) indicates the deviation of the estimated DOA from the true DOA. The result of RMSE is exhibited in Fig. 4, where the SNR varies from $-5$ dB to 20 dB. Each point in Fig. 4 is achieved by 500 independent Monte Carlo runs, and 5 signals are considered. It can be seen that EBSA always outperforms others in terms of estimation accuracy for the full range of SNR. The final simulation investigates the change of RMSE with the number of samples and is shown in Fig. 5. In this simulation, 5 signals with SNR of 5 dB are considered, and the initial number of samples is 50. As shown in Fig. 5, EBSA can achieve better estimation accuracy than H-MUSIC, HDAPA and BSA.

## Conclusion

EBSA based on the hybrid analog-digital structure with nested array is proposed in this paper. By eliminating the redundant calculations for solving the linear equations, the computational cost in reconstructing the SCM is greatly reduced. Also, based on the hybrid analog-digital structure, the proposed EBSA with nested array can resolve more sources than BSA with uniform linear array, owing to the advantage of nested array in providing high degrees of freedoms. Finally, simulation experiments are performed to verify the performance of EBSA in the computational cost and DOA estimation performance. By comparing EBSA with three recently proposed DOA estimation algorithms for hybrid analog-digital structure, the results indicate that the EBSA always achieves better RMSE performance than other algorithms during all the regions of SNR and samples.
